# Clinical Characteristics and Visual Outcomes in Patients with Intralenticular Foreign Bodies with Self-Sealing Corneal Penetrating Wounds

**DOI:** 10.1155/2021/6613205

**Published:** 2021-06-21

**Authors:** Zhitao Su, Yuanqi Wang, Quanyong Yi, Lin Lin, Kairan Lai, Panpan Ye, Yao Wang, Xiaoyun Fang

**Affiliations:** ^1^Eye Center, Second Affiliated Hospital, School of Medicine, Zhejiang University, Hangzhou 310009, China; ^2^Eye Hospital, Zhejiang University, Hangzhou 310009, China; ^3^Ningbo Eye Hospital, Ningbo 315040, China

## Abstract

**Purpose:**

Siderosis bulbi may occur as a result of retained intralenticular foreign bodies (ILFBs) that were missed during examination in patients with self-sealing wounds and without a significant decrease in visual acuity. This study aimed to explore the clinical characteristics and visual outcomes of ILFBs with self-sealing corneal penetrating wounds.

**Methods:**

Fifteen eyes of 15 patients with ILFBs and self-sealing corneal penetrating wounds, seen between October 2014 and September 2019, were retrospectively analyzed. Data regarding the patient demographics, clinical features, surgical procedure, and initial and final best-corrected visual acuity (BCVA) were analyzed.

**Results:**

All patients were male with a mean age of 41 years. The foreign bodies passed through the cornea, sometimes through the iris, through the anterior capsule, and finally localized in the lens. All ILFBs were pointed and metallic objects and were successfully removed with phacoemulsification and posterior chamber intraocular lens (IOL) implantation. Anterior capsule violation was found in three eyes, but no posterior capsule rupture was found. The IOL was placed in a capsule bag in all the cases. The BCVA ranged from 20/200 to 20/25 preoperatively and improved to between 20/32 and 20/20 at the last follow-up visit. The IOLs were well-centered. Apart from posterior capsule opacity in four eyes, no other postoperative complications were found.

**Conclusions:**

In patients with a pointed metallic ILFB and self-sealing corneal penetrating wounds (with or without cataracts), early diagnosis and removal of the metallic ILFB combined with lens removal and IOL implantation may avoid late complications and achieve good visual outcomes.

## 1. Introduction

Ocular trauma is a major cause of ocular morbidity in the working population [1, 2]. Without appropriate diagnosis and treatment, penetrating ocular injury with an intraocular foreign body (IOFB) can lead to blindness or other severe ocular complications [3]. Injuries to the anterior and posterior segments may occur due to ocular trauma, and surgical treatment should be undertaken to reconstruct the anterior and posterior segments [4–8]. Siderosis bulbi, one of the most serious complications, may be caused by retention of an iron-containing IOFB, which can cause deposition of iron molecules in the ocular tissues [9, 10]. The clinical findings of siderosis bulbi include iris heterochromia, pupillary mydriasis, cataract formation, secondary glaucoma, retinal arteriolar narrowing, retinal pigmentary degeneration, optic disc swelling or hyperemia, and cystoid macular edema [9–12]. Without a complete examination, intralenticular foreign bodies (ILFBs) may be missed and therefore retained in patients with small self-sealing wounds who present with no decreased visual acuity [13, 14]. Furthermore, reports on patients with siderosis bulbi caused by retention of an ILFB are rare [15, 16]. Here, we report a series of 15 eyes with metallic ILFBs and a self-sealing corneal wound. Patient demographics, clinical features, nature of the foreign body, surgical procedure, and initial and final best-corrected visual acuity (BCVA) were analyzed.

## 2. Materials and Methods

The study involved 15 eyes of 15 patients with self-sealing corneal penetrating wounds and ILFBs, with or without traumatic cataract, who were seen between October 2014 and September 2019. Eyes with endophthalmitis, retinal injury, vitreous hemorrhage, or an IOFB in the posterior segment were excluded. This study followed the tenets of the Declaration of Helsinki and was approved by the Ethics Committee of the Second Affiliated Hospital, School of Medicine, Zhejiang University. Written informed consent was obtained from all patients.

A thorough history was obtained from each patient. BCVA, slit-lamp examination with pupil dilation, and binocular indirect ophthalmoscopy were performed. Whenever possible, B-scan ultrasonography or orbital computerized tomography scanning was performed to evaluate the eye injury. After identification of no other foreign body apart from the ILFB, the lens and the ILFB were removed and intraocular lens (IOL) implantation was performed with or without corneal suturing.

Before surgery, the pupil was dilated with compound tropicamide eye drops. All patients received retrobulbar anesthesia with injections of 2% lidocaine and 0.75% bupivacaine. Viscoelastics were injected into the anterior chamber through the side incision, and corneal suturing was performed if necessary. After division of the iris posterior synechiae by the viscoelastics, continuous curvilinear capsulorhexis was performed from the anterior penetrating wound caused by the ILFB. The lens material was removed before the ILFB by phacoemulsification or aspiration, and the ILFB was then removed using forceps through the main incision. The residual lens material was removed by phacoemulsification or aspiration, followed by IOL implantation in the capsule bag (11 implants were AMO Tecnis ZCB00 (Johnson & Johnson Surgical Vision, CA, USA) and four were Akreos Adapt AO (Bausch & Lomb, NY, USA)). To prevent enlargement and violation of the anterior capsule in three eyes with incomplete continuous curvilinear capsulorhexis caused by the peripheral ILFB, the bottle height was set to 80 cm, the maximal vacuum was set to 250 mmHg, and viscoelastics were injected into the anterior chamber before withdrawal of the ultrasonic or irrigation/aspiration handle. Tobramycin-dexamethasone eye ointment was applied to the conjunctival sac at the end of surgery.

After surgery, the patient was administered 0.5% levofloxacin eye drops, 1% prednisolone acetate eye drops, and 1% pranoprofen eye drops 2–8 times a day for 4 weeks and a levofloxacin tablet (0.5 g) daily for 7 days. Patients were followed up at 1 day, 1 week, 3 months, and 6 months postoperatively. Slit-lamp examination, intraocular pressure, fundus examination, and BCVA were assessed.

## 3. Results

Fifteen patients were included in the analysis. All patients were men. Most injuries occurred as a result of the hammering of metals during occupational activities.  Table 1 presents the patient characteristics and visual outcomes. The average age of the patients was 41 years (range, 22–55 years). The right eye was injured in six patients and the left eye in nine patients. The foreign bodies passed through the cornea, the iris in some patients, the anterior capsule, and finally localized in the lens. Twelve cases presented with paracentral self-sealing corneal penetrating wounds, and three cases presented with peripheral self-sealing corneal penetrating wounds. Iris posterior synechiae was found in six eyes, iris defect in six eyes, and no iris damage in three eyes. Twelve eyes developed localized cataracts, and three eyes showed no sign of cataracts. The average time interval between injury and surgery was 19 days (range: 1–89 days). Corneal suturing was performed in 11 eyes. All foreign bodies were successfully removed through incision of the main cornea. The mean size of the ILFBs was 1.5 mm (range: 1.0–2.0 mm) in width and 2.5 mm (range: 1.5–4.0 mm) in length.

**Figure 1:**
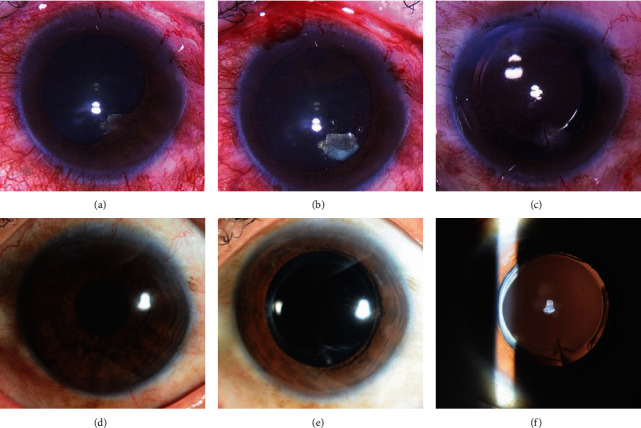
Patient 1 with iris posterior synechiae and corneal suture. (a) Anterior segment photograph revealing a paracentral self-sealing corneal penetrating wound at the 5 o'clock position, iris posterior synechiae, and part of a metallic-like ILFB after pupil dilation. (b) A metallic-like foreign body identified after division of the iris posterior synechiae during surgery. (c) Lens material and ILFB removal is combined with IOL implantation and corneal wound suturing. (d)–(f) Anterior segment photographs show the round pupil and well-centered IOL 3 months after surgery. ILFB, intralenticular foreign body; IOL, intraocular lens.

Figure 1 shows a patient with iris posterior synechiae and localized cataracts (patient 1). A paracentral self-sealing corneal penetrating wound, iris posterior synechiae, and part of a metallic-like foreign body were found in the 5 o'clock position by slit-lamp examination after pupil dilation (Figure 1(a)). After iris posterior synechiae division during surgery, a metallic foreign body (2.0 mm × 3.0 mm) was observed (Figure 1(b)). Corneal suturing, ILFB removal, cataract removal, and IOL implantation were successfully performed (Figure 1(c)). Three months postoperatively, the IOL was well-centered, and the BCVA was 20/20 (Figures 1(d)–1(f)).

**Figure 2:**
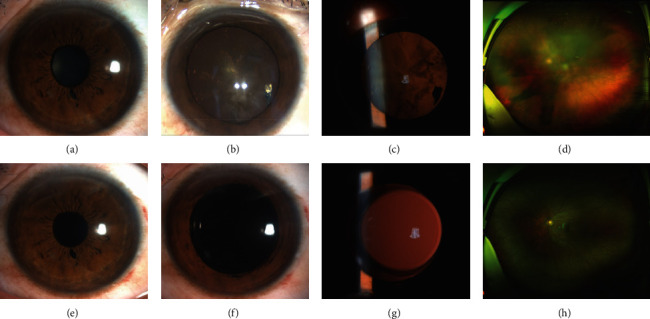
Patient 10 with iris defect and localized cataract. (a) Anterior segment photograph revealing a peripheral self-sealing corneal penetrating wound at the 7 o'clock position and an iris defect at the 6 o'clock position. (b)-(c) A metallic-like foreign body and localized cataract identified after pupil dilation. (d) A scanning laser ophthalmoscopic image showing a shadow caused by the traumatic cataract. (e)–(g) Anterior segment photographs showing the round pupil with the iris defect and the well-centered IOL 3 months after surgery. (h) Retinal arteriolar narrowing, pigmentary retinal degeneration, optic disc swelling, and cystoid macular edema not observed on the scanning laser ophthalmoscopic image. IOL, intraocular lens.

Figure 2 presents a patient with an iris defect and localized cataract (patient 10). A paracentral self-sealing corneal penetrating wound at the 7 o'clock position and iris defect adjacent to the pupil margin at the 6 o'clock position were found by slit-lamp examination without pupil dilation (Figure 2(a)). A small metallic-like foreign body (1.0 mm × 1.5 mm), localized in the lens at the 4 o'clock position, and traumatic cataracts were observed after pupil dilation (Figures 2(b) and 2(c)). A scanning laser ophthalmoscopic image showed a shadow caused by a traumatic cataract (Figure 2(d)). Removal of the ILFB and cataracts and IOL implantation were successfully performed without corneal suturing. At the final follow-up, the IOL was well-centered, and the BCVA was 20/20 (Figures 2(e)–2(g)) without retinal arteriolar narrowing, pigmentary retinal degeneration, optic disc swelling, or cystoid macular edema (Figure 2(h)).

**Figure 3:**
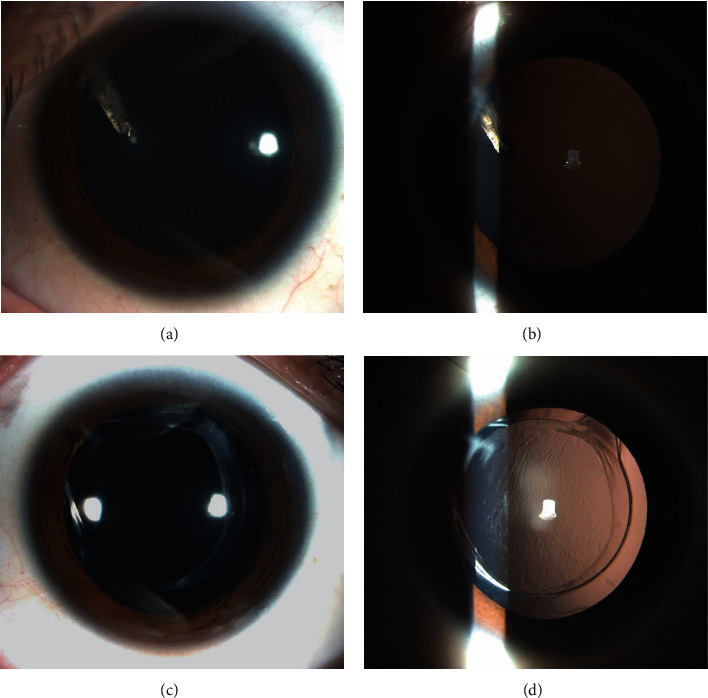
Patient 13 with clear lens and anterior capsule violation. (a)-(b) Anterior segment photographs revealing a peripheral self-sealing corneal penetrating wound at the 10 o'clock position, iris margin injury, a metallic-like foreign body, and clear lens after pupil dilation. (c)-(d) Anterior segment photographs showing anterior capsule violation and the well-centered IOL 3 months after surgery. IOL, intraocular lens.

Figure 3 shows a patient with ILFB and a clear lens (patient 13). A peripheral self-sealing corneal penetrating wound at the 10 o'clock position, iris margin damage, and a metallic-like foreign body (1.5 mm × 2.5 mm) localized in the lens without cataract at the 10 o'clock position were observed after pupil dilation (Figures 3(a) and 3(b)). Removal of the ILFB and the lens and IOL implantation were successfully performed without corneal suturing. At the final follow-up, the IOL was well-centered, and the BCVA was 20/20 (Figures 3(c) and 3(d)).

The mean follow-up time for all patients was 10 months (range: 6–18 months). Incomplete continuous curvilinear capsulorhexis was found in three eyes with anterior capsule violation caused by the peripheral ILFB. The IOL was placed in a capsule bag in all cases. The BCVA ranged from 20/200 to 20/25 preoperatively and improved to 20/32 to 20/20 at the last follow-up visit. The IOL was well-centered in all eyes. Posterior capsule opacification was observed in four eyes. Other complications, such as secondary glaucoma, endophthalmitis, or siderosis bulbi, were not observed.

## 4. Discussion

ILFBs constitute 5–10% of all IOFBs and may have a more benign sequela than other IOFBs [17]. Various types of foreign bodies in the lens have been reported, including glass [18], eye lashes [19], wood, stone, and metal [20]. The management of an ILFB includes an assessment of its material, size, location, potential for infection, lenticular damage degree, and degree of damage to other related tissues [21]. This study involved a series of 15 eyes with ILFB and a self-sealing corneal wound. The foreign bodies were pointed and metallic. They passed through the cornea, sometimes through the iris, then through the anterior capsule, and finally localized in the lens. Removal of the ILFB and lens material and IOL implantation were successfully performed in all cases. BCVA after surgery was equal to or better than 20/32.

When an IOFB cannot be found during ophthalmological evaluation, tools, including plain radiography, ultrasonography, ultrasound biomicroscopy, orbital computerized tomography scanning, magnetic resonance imaging, and optical coherence tomography, are available to aid in IOFB diagnosis [11, 22]. Ultrasonography and computerized tomography are more sensitive methods for detecting all types of IOFBs [23]. In this study, ILFB diagnosis was missed in three eyes with impaired vision at the primary visit where pupil dilation was not performed. Patients visited our eye center because of reduced visual acuity caused by progressive traumatic cataract. Any patient presenting with penetrating ocular injury should be suspected of having IOFB and should be followed up closely. Indications of a possible IOFB include small self-sealing wounds, iris transillumination defects, iris heterochromia, irregular pupils, and focal lens opacities [24].

There is no consensus on whether ILFBs should be removed. Evaluation of ILFBs and any associated injury is necessary to determine the best approach. If the lens damage is localized and does not involve the visual axis and the foreign body is inert and nonmagnetic, the best policy is to wait and let the foreign body remain in situ [13]. Stable visual function without significant cataract formation has been described in some cases [25, 26]. If visual acuity is compromised by cataract formation induced by the ILFB, removal of the cataract and ILFB is necessary [27]. All iron foreign bodies should be removed as early as possible to avoid siderosis bulbi, even if metallic ILFBs do not involve the visual axis [25]. Considering the impossibility of close follow-up and the potential for siderosis bulbi, removal of the metallic ILFB and clear lens and IOL implantation were performed in three eyes in this study. Once the decision to remove the ILFB is made, surgery should be performed as early as possible. In this study, the time interval between injury and surgery was 19 days on average (range: 1–89 days), and all the ILFBs were removed 3 days after detection.

The procedure for removal of ILFBs has changed often over the last century. Initially, intracapsular cataract extraction was advocated [28, 29]. After the 1930s, popular procedures involved removal of IFLBs by manipulating them into the anterior chamber, either manually or with the assistance of a magnet and then removing them through the original corneal entry site or via a separate surgical incision [30]. Extracapsular cataract extraction and IOL implantation, combined with extraction of magnetic ILFBs, have been reported to be successful [31, 32]. Advancements in microincision phacoemulsification, removal of ILFBs, phacoemulsification, and IOL implantation (tricombined operation) have been well reported [27, 33]. It is important to locate the ILFB and determine whether the posterior capsule is intact before surgery [34]. Particles of the ILFB or lens material may be deposited, and vitreous prolapse may occur in the eyes with posterior capsule rupture without proper maneuvering. Anterior vitrectomy should be performed to remove the vitreous in the anterior chamber [35]. IOL fixation techniques may be used in eyes without sufficient capsular support [36, 37]. In this study, a tricombined operation was successfully performed, no posterior capsule rupture occurred, and the IOL was placed in the capsule bag in all cases.

Previous studies have reported that posterior segment IOFBs are usually associated with more complicated conditions, such as retinal detachment, endophthalmitis, proliferative vitreoretinopathy, and an epiretinal membrane, and the eyes with these conditions are severely damaged with a relatively poor final BCVA [38, 39]. An anterior IOFB is usually associated with a better final BCVA than a posterior IOFB. In the present study, the final BCVA was equal to or better than 20/32. Reasons for a good visual outcome lie in certain injury-related characteristics. First, some foreign bodies were embedded in the lens and isolated from other ocular tissues; however, some foreign bodies were exposed in the anterior chamber, and because of the short duration between injury and surgery, ILFBs did not cause any inflammation or toxic reactions. Second, the self-sealing wounds were limited to the paracentral or peripheral cornea, resulting in no significant astigmatism. Third, even when a cataract forms and vision deteriorates, modern advances in cataract surgery techniques mean that lens replacement is a viable and likely very successful option.

## 5. Conclusions

In summary, a pointed metallic ILFB may cause self-sealing corneal penetrating wounds, with or without cataracts. Early diagnosis and removal of the metallic ILFB, combined with lens removal and IOL implantation, may avoid late complications and result in good visual outcomes.

## Figures and Tables

**Table 1:** Clinical characteristics and visual outcomes of all patients.

No.	Age (yrs)	Eye	Cornea entry site	Iris injury	Cataract	Time to surgery (ds)	Corneal suture	Size of FB (mm)	Follow-up (ms)	Preoperative		Final	
										Refraction	BCVA	Refraction	BCVA
1	49	Right	PC	IPS	Localized	4	Yes	2.0 × 3.0	6	−1.00 − 2.75 × 130	20/32	−0.50 − 1.25 × 145	20/20
2	50	Left	PC	IPS	Localized	10	Yes	2.0 × 2.5	12	−1.50 − 2.50 × 45	20/63	−0.50 − 1.25 × 60	20/32
3	40	Right	PC	Nil	Localized	50	Nil	1.0 × 1.5	6	−0.50 − 1.50 × 70	20/200	−2.00 − 1.00 × 65	20/25
4	30	Left	PC	ID	Localized	15	Yes	2.0 × 3.0	18	−2.00 − 4.00 × 135	20/63	−0.75 − 2.50 × 145	20/25
5	31	Left	PC	IPS	Localized	26	Yes	1.5 × 2.0	15	−3.00 − 2.00 × 65	20/125	−2.00 − 1.25 × 50	20/32
6	55	Left	PC	IPS	Localized	10	Yes	2.0 × 2.5	9	−0.50 − 3.00 × 140	20/63	−0.75 − 1.50 × 130	20/32
7	32	Right	P	ID	Nil	2	Yes	1.0 × 2.0	18	−4.00 − 1.75 × 85	20/25	−3.00 − 1.00 × 70	20/20
8	51	Left	P	ID	Localized	3	Yes	1.0 × 2.5	6	−1.00 − 3.50 × 45	20/80	−0.75 − 2.00 × 55	20/25
9	38	Right	PC	Nil	Nil	21	Nil	1.0 × 2.0	6	−2.00 − 1.50 × 60	20/32	−1.75 − 1.25 × 63	20/20
10	42	Left	PC	ID	Localized	89	Nil	1.0 × 1.5	12	−2.50 − 1.00 × 145	20/100	−0.25 − 0.50 × 155	20/20
11	46	Left	PC	IPS	Localized	7	Yes	2.0 × 2.5	12	−3.00 − 3.00 × 150	20/63	−2.00 − 1.50 × 170	20/20
12	48	Right	PC	ID	Localized	2	Yes	1.0 × 4.0	6	−0.25 − 3.50 × 70	20/80	−0.50 − 2.50 × 50	20/32
13	22	Left	P	IPS	Nil	5	Yes	1.5 × 2.5	12	−1.25 − 1.75 × 35	20/25	−2.00 − 1.00 × 27	20/20
14	38	Right	PC	ID	Localized	1	Yes	1.5 × 3.0	12	−0.75 − 4.50 × 55	20/100	−0.50 − 2.50 × 60	20/25
15	46	Left	PC	Nil	Localized	35	Nil	1.5 × 2.5	6	−1.00 − 2.50 × 155	20/125	−0.50 − 1.50 × 140	20/25

yrs, years; PC, paracentral; P, peripheral; IPS, iris posterior synechiae; ID, iris defect; ds, days; FB, foreign body; ms, months; BCVA, best-corrected visual acuity.

## Data Availability

The data used to support the finding of this study are available from the corresponding author upon request.

## References

[1] Bord S. P., Linden J. (2008). Trauma to the globe and orbit. *Emergency Medicine Clinics of North America*.

[2] Hollander D. A., Aldave A. J. (2002). Ocular bungee cord injuries. *Current Opinion in Ophthalmology*.

[3] Ehlers J. P., Kunimoto D. Y., Ittoop S., Maguire J. I., Ho A. C., Regillo C. D. (2008). Metallic intraocular foreign bodies: characteristics, interventions, and prognostic factors for visual outcome and globe survival. *American Journal of Ophthalmology*.

[4] Frisina R., Parrozzani R., Tozzi L., Pilotto E., Midena E. (2020). Pupil cerclage technique for treatment of traumatic mydriasis. *European Journal of Ophthalmology*.

[5] Frisina R., De Biasi C. S., Londei D., Gambato C., Midena E. (2020). A new intraocular lens with artificial iris for treating a case of iris extrusion secondary to traumatic opening of a radial keratotomy. *European Journal of Ophthalmology*.

[6] Frisina R., Pilotto E., Tozzi L., Parrozzani R., Midena E. (2019). A new technique of needle-guided retropupillary fixation of iris-claw intraocular lens. *Journal of Cataract and Refractive Surgery*.

[7] Gurler B., Coskun E., Oner V., Comez A., Erbagci I. (2017). Syrian civil-war-related intraocular foreign body injuries: a four-year retrospective analysis. *Seminars in Ophthalmology*.

[8] Assi A., Chacra C. B., Cherfan G. (2008). Combined lensectomy, vitrectomy, and primary intraocular lens implantation in patients with traumatic eye injury. *International Ophthalmology*.

[9] Appel I., Barishak Y. R. (1978). Histopathological changes in siderosis bulbi. *Ophthalmologica*.

[10] Tawara A. (1986). Transformation and cytotoxicity of iron in siderosis bulbi. *Investigative Ophthalmology & Visual Science*.

[11] Casini G., Sartini F., Loiudice P., Benini G., Menchini M. (2021). Ocular siderosis: a misdiagnosed cause of visual loss due to ferrous intraocular foreign bodies-epidemiology, pathogenesis, clinical signs, imaging and available treatment options. *Documenta Ophthalmologica*.

[12] Xu Z., Zhong J. M., Ye P. P. (2020). Resolution of siderotic glaucoma correlated with decreased pigmentation in the anterior chamber angle after removal of a retained ferrous foreign body. *International Journal of Ophthalmology*.

[13] Chang Y. S., Jeong Y. C., Ko B. Y. (2008). A case of an asymptomatic intralenticular foreign body. *Korean Journal of Ophthalmology*.

[14] Foss A. J., Forbes J. E., Morgan J. (1993). An intralenticular foreign body and a clear lens. *British Journal of Ophthalmology*.

[15] Wu T.-T., Kung Y.-H., Sheu S.-J., Yang C.-A. (2009). Lens siderosis resulting from a tiny missed intralenticular foreign body. *Journal of the Chinese Medical Association*.

[16] O’Duffy D., Salmon J. F. (1999). Siderosis bulbi resulting from an intralenticular foreign body. *American Journal of Ophthalmology*.

[17] Coleman D. J., Lucas B. C., Rondeau M. J., Chang S. (1987). Management of intraocular foreign bodies. *Ophthalmology*.

[18] Hassan N. A., Reddy M. A., Reddy S. S. (2009). Late occurrence of lens particle glaucoma due to an occult glass intralenticular foreign body. *Middle East African Journal of Ophthalmology*.

[19] Byrnes V. A. (1949). Eyelash buried in clear lens substance. *American Journal of Ophthalmology*.

[20] Bai H.-Q., Yao L., Meng X.-X., Wang Y.-X., Wang D.-B. (2011). Visual outcome following intraocular foreign bodies: a retrospective review of 5 year clinical experience. *European Journal of Ophthalmology*.

[21] Reddy S. C. (2011). Intralenticular metallic foreign body: a case report. *International Journal of Ophthalmology*.

[22] Güler M., Yilmaz T., Yigit M., Ulku G., Arslan S. (2010). A case of a retained intralenticular foreign body for two years. *Clinical Ophthalmology*.

[23] Arnáiz J., Marco de Lucas E., Piedra T. (2006). Intralenticular intraocular foreign body after stone impact: CT and US findings. *Emergency Radiology*.

[24] Su Z., Yin H., Ye P. (2019). Pterygium surgery combined with the removal of a missed occult iris foreign body detected incidentally during pterygium examination: a case report. *BMC Ophthalmology*.

[25] Keeney A. H. (1971). Intralenticular foreign bodies. *Archives of Ophthalmology*.

[26] Pieramici D. J., Capone A., Rubsamen P. E., Roseman R. L. (1996). Lens preservation after intraocular foreign body injuries. *Ophthalmology*.

[27] Han S., Wang T., Jia J. (2019). Visual outcomes and prognostic factors of intralenticular foreign bodies in a tertiary hospital in North China. *Journal of Ophthalmology*.

[28] Donaldson D. D. (1965). Intralenticular foreign body. *Archives of Ophthalmology*.

[29] Ide C. H. (1968). Metallic foreign body in lens twenty-two years. *American Journal of Ophthalmology*.

[30] Galin M. A., Taylor A., McLean J. M. (1961). Intralenticular foreign bodies. *Archives of Ophthalmology*.

[31] Bishara S. A., Nesher R. G., Zelikovitch A. (1985). Extracapsular extraction and lens implantation for cataracts with foreign bodies. *Annals of Ophthalmology*.

[32] Macken P. L., Boyd S. R., Feldman F., Heathcote J. G., Steiner M., Billson F. A. (1995). Intralenticular foreign bodies: case reports and surgical review. *Ophthalmic Surgery, Lasers and Imaging Retina*.

[33] Mester V., Kuhn F. (2002). Intraocular foreign bodies. *Ophthalmology Clinics of North America*.

[34] Singh R., Ram J., Gupta R. (2015). Use of Scheimpflug imaging in the management of intra-lenticular foreign body. *Nepalese Journal of Ophthalmology*.

[35] Vajpayee R. B., Sharma N., Dada T., Gupta V., Kumar A., Dada V. K. (2001). Management of posterior capsule tears. *Survey of Ophthalmology*.

[36] Czajka M. P., Frajdenberg A., Stopa M., Pabin T., Johansson B., Jakobsson G. (2020). Sutureless intrascleral fixation using different three‐piece posterior chamber intraocular lenses: a literature review of surgical techniques in cases of insufficient capsular support and a retrospective multicentre study. *Acta Ophthalmologica*.

[37] Saleh M., Heitz A., Bourcier T. (2013). Sutureless intrascleral intraocular lens implantation after ocular trauma. *Journal of Cataract and Refractive Surgery*.

[38] Nicoara S. D., Irimescu I., Calinici T., Cristian C. (2015). Intraocular foreign bodies extracted by pars plana vitrectomy: clinical characteristics, management, outcomes and prognostic factors. *BMC Ophthalmology*.

[39] Yang C.-S., Hsieh M.-H., Hou T.-Y. (2019). Predictive factors of visual outcome in posterior segment intraocular foreign body. *Journal of the Chinese Medical Association*.

